# Antiseptic Action of Hypochlorous Acid and Sodium Hypochlorite

**Published:** 1916-04

**Authors:** M. C. Pruitt

**Affiliations:** 824 Healy Bldg., Atlanta, Ga., U. S. A.


### ANTISEPTIC ACTION OF HYPOCHLOROUS ACID AND SODIUM HYPOCHLORITE.

By M. C. Pruitt, L. R. C. P., L· R. S. P., L. R F P S MD

(As noted in civilian and military wards since August, 1915).

The primary object of an ideal antiseptic is one which possesses the power of rapidly destroying spores as well as the ordinary bacterial forms and does not produce pain or irritation, and is non-toxic to the tissue. With this object in view we are continually changing from one to another; today one antiseptic is in the front line of trenches and tomorrow another may be. But at present from observation both in civilian and military wards of Westminster Hospital London, England, since August, 1915, to date I am convinced that Sodium Hypochlorite and Hypochlorous Acid are in the front line.

### Preparation of Mixtures

### Sodium Hypochlorite.

Prepared as follows:

140 grams of dry sodium carbonate or 400 grams of crystallized salt is dissolved in 10 litres of tap water, and 200 grams of chlorinated lime of good quality added. The mixture is well shaken, allowed to stand, and the clear liquid syphoned off and filtered through a plug of cotton. To this is added 40 grams of boric acid, and, when disolved, the solution is ready for use. The solution should not be kept longer than one week, and it is important to note that the boric acid should not be added to the mixture before filtering, but afterwards. A slight additional precipitate of calcium salts may slowly occur, after filtering the mixture, but this is of no significance (H. D. Dakin, Brit. Med. Journal, August 28th, 1915, 318).

### Hypochlorous Acid

Prepared as follows:

Ordinary commercial bleaching powder or chloride of lime is ground in a mortar to a fine powder, and then equal weight mixed with boric acid powder.

Solution may be prepared by two methods:

1. 25 grams of this mixture are shaken up with one litre of water, allowed to stand for a few hours, then filter through cloth or filter paper.

2. To one litre of water add 12.5 grammes of bleaching powder, shake vigorously, then add 12.5 grams of boric acid powder and shake again. Allow to stand for some hours, preferably over night, then filter off, and the clear solution is ready for use.

This solution contains:—

| | |
|---|---|
| Hypochlorous Acid | 0.54 per cent. |
| Calcium Biborate | 1·28 per cent. |
| Calcium Chloride | .17 per cent. |
| Total | 1.99 per cent. |

(Smith Drennan Rettie & Campbell, Brit. Med. Journal, July 24, 1915.)

### Observations Military.

1st. About 98 per cent· of all wounds that reach the London Base Hospitals are infected with a mixed chain of bacteria. 2nd. A large per cent contain bullets, pieces of shrapnel, fragments of clothes, and other foreign bodies thus: One case I removed a piece of rubber roofing about 1 1-2 by 2 inches from the subscapula fossa.

The first object in the treatment is to remove the foreign bodies and make ample drainage. The second object is to find a cleaning agent with such bacteriocidal powers that it will act in the presence of serum, pus, blood or any of the secretions or execretions of the body and not be destructive to tissue; but on the contrary, be a stimulant to repair without causing pain or irritation. No one drug or antiseptic has ever filled these necessary factors. So we content ourselves by using the one we think best and continue to try to find a better or more efficient one, hence Hypochlorous Acid and Sodium Hypochloride.

### Type of Cases

G. S. W. Septic of almost every part of the body, thus: septic gunshot wound or frontal sinus and the bridge of nose. Fourteen days standing when admitted to the hospital; had had a previous operation on the second day after receiving the wound, in a Base hospital in France. Drainage was ample, very much necrosis, a profuse purulent discharge and a foul odor. Patient's wound was irrigated four hourly, one in ten sodium hypochlorite solution, clean in ten days.

2. Septic GSW of eye and inferior part of orbit was destroyed. Patient had an enucleation in France 14 days before admittance to Westminster. Large amount of pus and necrosis; clean on seventh day. Irrigation.

3. Septic GSW of almost any part of extremities and almost any complication. After ample drainage and removal of foreign bodies usually clean from three to fifteen days. Compound fractures of femur from shrapnel with multiple pieces of shrapnel and shattered bone which cannot easily be removed and an exception to this rule usually requiring drainage from one to three months.

4. Appendiceal abcess. Drained, slight amount of fecal discharge for first three days, irrigated with one in ten Hypochlorous Acid solution six hourly, clean on 8th day, healed on 20th day, left hospital on 21st day. Other cases similar results.

6. Carcinoma of rectum, inoperable, three profuse hemorrhages in last fortnight before admission, large amount of mucus in stools and a very offensive odor; colotomy was done; the rectum was then irrigated through the colotomy opening; odor, mucus and hemorrhage cleared up after third day—irrigation, once daily, one in 20 hypochlorous acid solution.

7. Excision of the tongue, (five cases) used as a mouth wash and gargle; mouth and wound healed almost with no infection and the raw surfaces in the mouth were clean and no odor.

8. Fracture of pelvis, rupture of uretha and extravasation of urine, infected· Ample drainage and repair of uretha; clean on tenth day, patient left hospital apparently well on 28th day, returned to hospital 21 days later with necrosis of pubis; opened, curretted, drained, irrigated, t. i. d. one in ten sodium hypochlorite solution, clean on 8th day. Healed, discharged from hospital on 28th day.

9. Inoperable carcinoma of cervix; hemorrhage very foul, irritating discharge; douche and tampons; daily. Irritation odor and discharge relieved on 7th day, hemorrhage not improved.

10. Septic ulcers, wounds, etc., usually clean up in from 3 to 21 days.

11. I have used both preparations in over 200 cases and in almost any kind of a septic wound or sinus, etc. One gets in a general hospital about equal results with above.

12. In the Casualty department for street accidents, etc., I have treated many cases of various kinds of wounds with these preparations and find them equally as effective as tincture of iodine, in clean wounds, with practically no pain at time of application.

METHODS OF USE

1. Hypochlorous Acid may be applied as a powder and then moistened with water.

2. (Both solutions) As a lotion—dilute from full strength to one in 20 with water.

3· As irrigation—dilute to one in 20 with water (frequency of irrigation from once daily to 2 hourly).

4. Fomentations—covered with waterproof.

5. Gauze wrung out of solution without waterproof.

6. As a bath full strength or dilute up to 1 in 20.

7. As a douche or tampon, dilution depends on irritation.

8. As a gargle or mouthwash· (very unpleasant to taste and smell, but removed immediately by washing out the mouth with water).

9. Intra-venous injection. Smith & Ritchie (Scotland) have successfully treated two cases of Septicemia by injecting intra-venous 100 cc. m. at 37 degrees centigrade the following solution: equal parts of chloride of lime and boric acid; 25 grams of this mixture; water, one litre, shake, 1st stand for a few hours, then filter; add common salt eight and five tenths grams. No toxic effect followed this injection.

10. Bladder. I have successfully treated one case of very virulent cystitis with one in 20 dilution of sodium hypochlorite solution daily· Patient complained of slight pain during irrigation.

### Comparison of Two Solutions.

Observations of the antiseptic properties from a clinical standpoint are about equal.

Sodium hypochlorite solution probably more likely to cause irritation (chiefly cutaneous).

Hypochlorous acid solution easiest prepared.

Both preparations should not be used after 7 to 10 days and should be kept in well stoppered bottles.

### Conclusions

1. Clinical observations confirm the conclusions arrived at by co-operative clinical and experimental tests of various investigators that sodium hypochlorite solution and hypochlorous acid solution are the most powerful antiseptics known.

2. Hypochlorous can be used either in a powder or solutions· Both powder and solutions while extremely destructive to organisms and their spores cause little or no harm to the tissues.

4· Fetor is rapidly eliminated.

5. Effect of solution is local and does not cause any toxicity, therefore, no danger apprehended from absorption.

6. Induces a flow of lymph from the wound as a part of the reaction of the tissues.

7. If pain or irritation occur can be easily controlled by diluting the solution.

SOME PRACTICAL CONSIDERATIONS

1. Extremely active bleaching agent and should therefore not be brought into contact with colored fabrics; if cloth is kept in the solution for some time its fabrics become brittle from the destruction of the texture. Any towels, etc., left in this solution should be rinsed in large quantities of water.

2. Corrosive to metal. Instruments, needles, etc., should be carefully treated afterwards, otherwise they rust.

3. Preparation inexpensive. Ingredients procurable everywhere. Preparing the solution a very simple process.

Present address: Resident Surgical Officer, Westminster Hospital, London, Eng.
Home address: 824 Healy Bldg., Atlanta, Ga., U. S. A.

